# Effect of Electrodeposition Conditions on Adsorption and Photocatalytic Properties of ZnO

**DOI:** 10.3390/ma18030497

**Published:** 2025-01-22

**Authors:** Alina Pruna, Iulian Poliac, David Busquets-Mataix, Antonio Ruotolo

**Affiliations:** 1 Center for Surface Science and Nanotechnology, National University of Science and Technology Politehnica Bucharest, 060042 Bucharest, Romania; 2 Institute of Materials Technology, Universitat Politècnica de València, 46022 Valencia, Spain; dbusquets@mcm.upv.es; 3 Gemmate Technologies, 10090 Buttigliera Alta, Turin, Italy; bogdan.poliac@gmail.com; 4 Department of Engineering, College of Charleston, Charleston, SC 29424, USA; ruotoloa@cofc.edu

**Keywords:** ZnO, graphene oxide, electrodeposition, adsorption, photocatalytic degradation

## Abstract

The electrodeposition of ZnO films was studied using potentiostatic mode in varying conditions including the presence of graphene oxide (GO) as a buffer layer and an additional deposition step. The obtained films were characterized by scanning electron microscopy (SEM), X-ray diffraction (XRD), Fourier transform Infrared (FTIR) spectroscopy, and X-ray photoelectron spectroscopy (XPS). The effect of electrodeposition conditions on the adsorption and photocatalytic properties of ZnO nanostructured films was analyzed by using methylene blue (MB) as a model dye molecule and exposure to UV light. The results indicated a marked effect of GO content in the buffer layer and the duration of nucleation on the properties of electrodeposited ZnO films. Lower GO content and an additional deposition step of 60 s resulted in the best adsorption and photocatalytic activity, these being 7 and 5-folds, respectively, in comparison to ZnO in absence of these adjustments. The MB photodegradation was found to follow first-order kinetics, the rate constant reaching a value of 2.38 × 10^−3^ min^−1^.

## 1. Introduction

Photocatalysis is one of the most common methods to remove organic dyes from residual water since it uses solar light as an energy source. As a photocatalyst, ZnO is considered as an alternative to the common TiO_2_ for dye removal from residual waters as it has a low cost, non-toxic nature and higher efficiency of light absorption [1]. However, ZnO performance is limited by the high recombination rate of the photo-generated charge carriers [2]. Varying approaches have been applied to overcome ZnO drawbacks [3]. One of the most common ones considers the morphology, namely that shapes such as rods have been indicated to improve photocatalytic degradation with respect to others like nanospindles or nanoflowers [4].

By coupling with other semiconductor oxides or by hybridizing with nanocarbons, the recombination of photo-induced charges may be achieved [5,6,7]. In this respect, graphene has been reported to act as a photoelectron trap, retarding the recombination of photo-induced charges’ separation, thereby improving the photoactivity of ZnO [6]. Graphene oxide (GO) is a derivative of graphene with high dispersibility in various solvents that helps simplify chemical hybridization methods [8]. The oxygen functional groups present on its surface have been exploited to grow ZnO [9]. Such groups also imply a reduction post-treatment to further improve the photocatalytic performance [10,11].

The synthesis of hybrids of ZnO with GO has been reported by varying methods. Amongst these, electrodeposition stands out due to its low cost, simplicity, low operation temperatures, and short process time [12]. Moreover, the size, shape, density, morphology, and crystallographic orientation of ZnO structures may be tailored by electrodeposition [13]. Although performing more poorly, an immobilized photocatalyst is considered preferable to a powder one as the recycling aspects may be improved [14]. An application for immobilized photocatalysts could be in reactors or textiles to degrade the dye from textile industry wastewaters. Electrodeposition presents an advantage in allowing the coating of complexly shaped substrates. Considering the need to reduce the GO, the electrochemical method was also reported to this end as displaying high efficiency, even in aqueous electrolytes [15].

While there are reports on the electrochemical deposition of ZnO on GO buffer layers, involving electroreduction in GO simultaneously with ZnO electrodeposition [10,16], there is still a lack of understanding due to non-homogeneity in the initial growth stage [17,18]. GO characteristics such as oxygen groups (content and distribution, oxidation/reduction degree) together with the versatility of electrodeposited ZnO allow for a multitude of conditions. To the best of our knowledge, there are no reports on the electrodeposition of ZnO films using potentiostatic mode in controlled conditions of nucleation duration and GO content in the buffer layer. In this work, the applicability of the electrodeposited ZnO films towards methylene blue removal under UV light of low irradiance was assessed. The obtained results indicate the optimum GO content and nucleation duration to enhance the MB removal performance in terms of degradation efficiency and rate constant.

## 2. Experiment

### 2.1. Materials

Analytic grade reagents (Sigma Aldrich, Fisher Scientific, Alcobendas, Spain) were used as received. Polyethylene terephthalate (PET) substrates (Sigma Aldrich) coated with Indium tin oxide (ITO) film (surface resistivity 60 Ω sq^−1^) were cleaned successively in an ultrasound bath in soapy water, distilled water, and isopropanol for 5 min each. Graphene oxide aqueous dispersion (2 mg mL^−1^, Sigma Aldrich, St. Louis, MO, USA) was employed to obtain diluted GO dispersions, namely 0.1 mg mL^−1^ and 0.25 mg mL^−1^. ITO substrate was dip-coated with 40 µL of GO dispersions with varying concentrations (0.1 and 0.25 mg mL^−1^) to obtain GO-coated ITO.

### 2.2. Electrodeposition of ZnO Nanostructured Coatings

Electrodeposition and electrochemical analyses were performed using a potentiostat (VersaSTAT 3, Princeton Applied Research, Beijing, China) in a classical three-electrode cell, with GO-coated ITO substrate, a Zn plate, and a saturated Ag/AgCl electrode serving as the working electrode, counter electrode, and reference electrode, respectively. The cell was maintained at 75 °C using a thermostatic bath. The ZnO films were electrodeposited from a 10 mM Zn(NO_3_)_2_ precursor electrolyte using potentiostatic mode at −1.05 V vs. Ag/AgCl for 600 s [19]. This condition is denominated as 1-step method. To improve the active surface area of ZnO, a 2-step method was designed by including a first potentiostatic step at a greater cathodic potential, namely −1.3 V, to improve the formation of nuclei. It was reported that potential values around −1.3 V were reported as the maximum to be employed for a reduction in GO in an aqueous electrolyte in order to avoid delamination of the GO film [15]. The duration of the nucleation step varied from 30 to 150 s.

### 2.3. Characterization

X-Ray diffraction (XRD) spectra were obtained using a D2 Phaser (Bruker) diffractometer (Beijing, China) employing a Cu Kα line of 1.54 Å, at 30 kV and 10 mA as the radiation source. The morphology was analyzed by using a scanning electron microscope (SEM, JSM-820 JEOL, Tokyo, Japan) working at 20 kV. The surface properties and chemical composition of the ZnO films were investigated using FTIR spectroscopy (Perkin Elmer, Waltham, MA, USA) in ATR mode and X-ray photoelectron spectroscopy (XPS) using a system (VG ESCA-LAB 220i-XL UHV) employing a monochromatic AlK X-ray source (1486.6 eV). The binding energy scale has been calibrated for surface charging by referencing the designated carbon C1s’ peak binding energy. Curve fitting has been performed by applying a Shirley-type background and deconvolution into various components with Gaussian fitting [20].

### 2.4. Photocatalytic Measurements

The photocatalytic activity of ZnO films under UV light was analyzed at 25 °C using a methylene blue (MB) model dye molecule and absorbance decay was measured with a Lambda 35 (Perkin Elmer, Waltham, MA, USA) spectrophotometer. To this end, the substrate (1 cm^2^ exposed area) was immersed horizontally in 10 mL aqueous solution of 1 µM MB and maintained in the dark for 30 min to establish an adsorption–desorption equilibrium of MB molecules on the surface of ZnO films. Next, the films were irradiated for 90 min with UV light (365 ± 15 nm, 4 W lamp, 0.4 mW cm^−2^ irradiance) located 10 cm above. At specific irradiation intervals, an aliquot of MB dye solution was taken and measured for absorbance decay at 665 nm at least twice. The degradation efficiency (*η*) of the MB dye was calculated as follows: *η*(%) = 100 ∗ (*A*_0_ − *A_t_*)/*A*_0_, where *A_0_* and *A_t_* are the absorbance intensity values of the MB dye at the initial and specified irradiation intervals, respectively. The photodegradation was fit to a pseudo-first-order kinetic model, and the apparent rate constant (*k*, min^−1^) was calculated from the slope of the −ln(*A_t_*/*A*_0_) = *k* ∗ *t*, where *t* is the employed duration [21].

## 3. Results and Discussion

**Figure 1:**
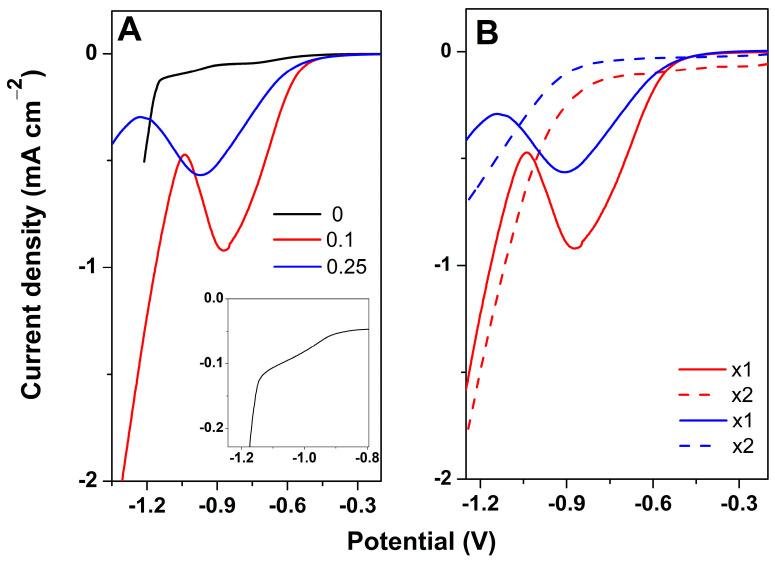
Cathodic polarization curves for the electrodeposition of ZnO as a function of GO concentration mg mL^−1^ (**A**). Two successive cathodic polarization curves for the electrodeposition of ZnO on GO 0.1 mg mL^−1^ and 0.25 mg mL^−1^ (**B**).

To investigate the reduction in GO layer together with the electrodeposition of ZnO, cathodic polarization curves were first recorded, as depicted in Figure 1. As observed in Figure 1A, the curve corresponding to absence of GO shows a small cathodic shoulder located at −1.05 V (see magnified inset image), attributed to ZnO electrodeposition. Such a finding is in high agreement with the Pourbaix diagram and other studies for the simultaneous electrodeposition of ZnO on the nanocarbon nucleation layer and the reduction in GO in aqueous solution [15].

In presence of a GO layer, a marked increase in the cathodic current is observed, being attributed to both ZnO deposition and GO reduction to reduced GO (rGO). The peak potential shifted to more negative values and there was a lower cathodic current at an increased GO content. This effect could be explained by the fact that oxygen functional groups decorating GO sheets act as nucleation sites to anchor the Zn^2+^ ions [22]. The lower peak intensity for a higher GO content indicates a hindering of the GO sheets from serving as a nucleation layer for ZnO due to agglomeration. To confirm the electro-reduction in GO sheets during the electrodeposition of ZnO, a second cathodic polarization curve was recorded, as depicted in Figure 1B. As observed, the second polarization curves are peak-free, indicating that the exposed oxygen functional groups of GO sheets were successfully removed.

**Figure 2:**
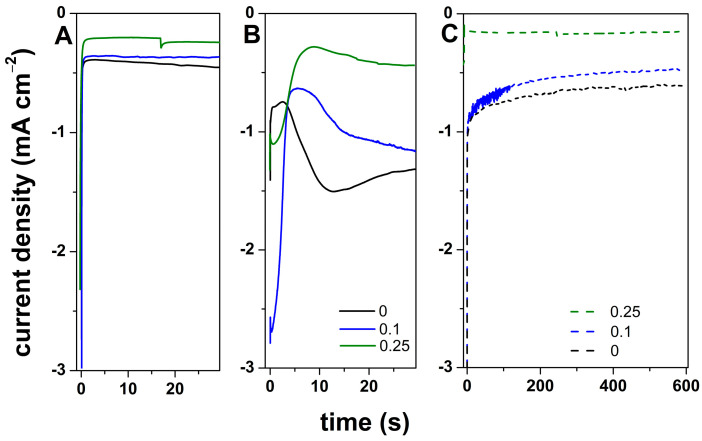
Chronoamperometric curves recorded for the electrodeposition of ZnO at −1.05 V (**A**), −1.3 V (**B**), and during the second deposition step (**C**) as a function of GO concentration mg mL^−1^.

In agreement with the polarization curves, the cathodic electrodeposition potential for ZnO was set to −1.05 V, while improved nuclei formation was considered by applying −1.3 V. The chronoamperometric curves recorded for the electrodeposition of ZnO onto ITO with GO content at −1.05 V are depicted in Figure 2A while those recorded at −1.3 V are depicted in Figure 2B. The comparison of the curves in Figure 2A,B show higher current density values for −1.3 V that clearly indicate an improved formation of nuclei and further growth. The peak that appeared in the first seconds in the curves in Figure 2B is attributed to GO reduction while the second peak is attributed to the formation of ZnO nuclei onto GO. The increase in GO content resulted in an increased duration to reach the nucleation current, as more functional groups in GO were available for this purpose. GO content also resulted in lower current density, which is indicative of lower-thickness film due to the presence of more nuclei. To confirm the effectiveness of the nucleation step, the curves recorded in the second deposition stage of the 2-step method (example shown after the nucleation for 30 s as depicted in Figure 2B) are further presented in Figure 2C. Since the deposition potential is the same, −1.05 V, the higher current density in Figure 2C, with respect to absence of nucleation step (Figure 2A), is due to the nucleation step, thus improving the formation of ZnO nuclei.

**Figure 3:**
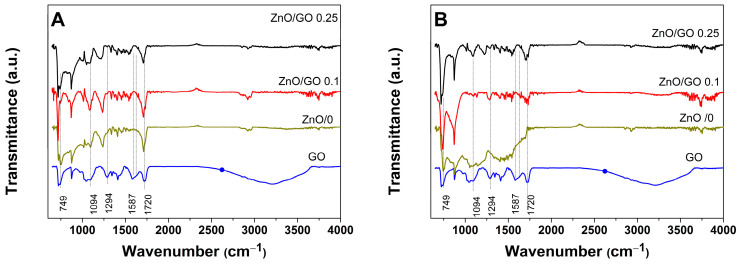
FTIR spectra of electrodeposited ZnO films with GO content by the 1-step method (**A**) and the 2-step one (**B**).

FTIR spectra of the electrodeposited ZnO as a function of the deposition steps and GO content are further depicted in Figure 3. The ITO substrate was employed to support all tested materials, and the spectrum of GO dispersion is depicted for reference. As it is observed, the peaks correspond to the ITO substrate such that at 871 cm^−1^ appears in the spectra. The FTIR spectrum of ZnO exhibits the characteristic signals located at 749 and 1544 cm^−1^ [23]. On the other hand, the GO spectra depicts typical peaks at 1724 cm^−1^, corresponding to the C=O stretching [24]; at 1623 cm^−1^, attributed to C=C stretching of aromatic ring; and the peaks at 1294 cm^−1^ and 1094 cm^−1^ corresponding to the C-O stretching [20]. The GO layer also exhibited a wide absorption band between 2600 and 3600 cm^−1^, corresponding to the stretching OH-groups’ vibrations, which indicates the presence of hydroxyl groups [25].

By comparing the spectra of ZnO electrodeposited in the absence and presence of GO content by the two methods, several changes were observed. Firstly, the intensity of the ITO peak at 871 cm^−1^ increased when deposition included a nucleation step and GO. Moreover, the highest intensity was obtained for the GO 0.1. A peak intensity increase was also observed for ZnO at 1544 cm^−1^ with GO content and a nucleation step. These changes indicate the improved density of smaller sized particles when the deposition includes a lower amount of GO and a nucleation step.

On the other hand, the comparison of GO peaks shows that these are either diminished (1724 cm^−1^) or even absent (1587 cm^−1^) in the hybrids, indicating the reduction in GO. The characteristic band of GO assigned to the stretching vibration of the O-H groups and centered at 3300 cm^−1^ was found absent in the hybrids and it may be attributed to the reduction and interaction of GO with the ZnO nanoparticles [23]. An improved electroreduction in GO and deposition of ZnO by the 2-step method is indicated by a lower intensity for the peak at 1094 cm^−1^ with respect to 1-step method. The interaction with ZnO through a GO reduction simultaneously with the formation and interaction with ZnO [26] is further indicated by peak shifts, such the peak at 1724 cm^−1^ corresponding to the C=O stretching that is diminished and shifted to 1730 cm^−1^ in the hybrids.

**Figure 4:**
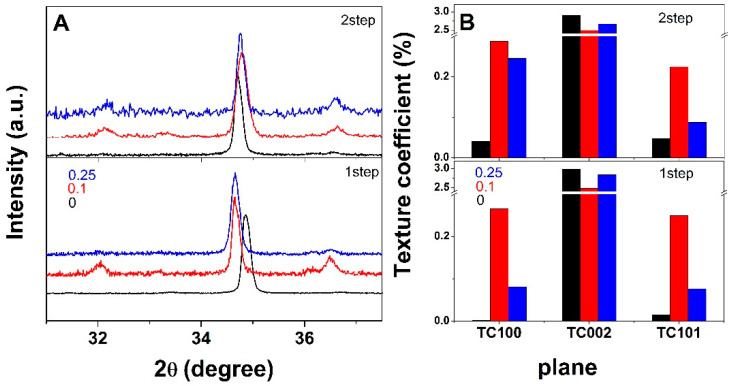
XRD spectra of ZnO films deposited as a function of method and GO content (**A**). Texture coefficient for the main peaks (**B**).

XRD was employed to analyze the crystal structure of electrodeposited ZnO, and the corresponding spectra are depicted in Figure 3. As the peaks exhibited very low intensity, except for the typical wurtzite hexagonal ZnO phase (JCPDS 36-1451) peaks (100), (002), (101) located at 2θ values of about 32.05, 34.7, 36.5, in Figure 4A only the angle range corresponding to these peaks is presented.

As can be observed in Figure 4A, the (002) peak exhibited the highest intensity, indicating c-axis preferential growth. The electrodeposition by the 1-step method of ZnO film onto GO resulted in a downshifted (002) peak, while in the case of the 2-step deposition method the shift is negligible. The intensity of the (100) and (101) planes increases in the presence of GO and by applying a nucleation step, attributed to the nucleation of ZnO induced by the oxygen functional groups in GO [11]. Simultaneously, the ITO peaks became observable, which indicated a thinner ZnO coating. The texture coefficient, *TC*, is further depicted in Figure 4B, where a value above 1 indicates a preferential orientation. While TC values for the (100) and (101) were below 1, these increased upon the use of GO and by applying a nucleation stage. Moreover, it is observed that the maximum values were obtained for lower content of GO. This result could be explained by the functional groups of GO serving as nucleation sites to anchor the Zn^2+^ ions [22,27], their availability being higher when lower content is employed while agglomeration occurs for higher GO content. On the other hand, a higher GO amount would result in agglomerated sheets that lead to a decrease in the availability of nucleation sites. The results also indicate a thinner film due to nuclei increasing in number rather than size [28]. The crystallite size was calculated by using the Scherrer equation D = Kλ/(β ∗ cosθ), where D is the crystallite size, K is the shape factor (K = 0.9), λ is the wavelength (1.54 Å), β is the full width at half-maximum intensity (FWHM), and θ is the Bragg diffraction angle. It is clear that the addition of a nucleation step reduces the crystallite size, namely from 55 nm to 35 nm, in agreement with TC values and other similar hybrids [29].

**Figure 5:**
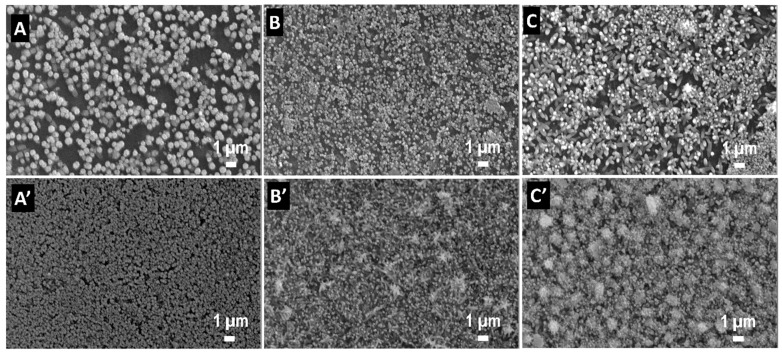
SEM images of the ZnO films electrodeposited by 1-step method (**A**–**C**) and 2-step method (**A’**–**C’**) as a function of GO content: 0 (**A**,**A’**), 0.1 (**B**,**B’**), and 0.25 (**C**,**C’**) mg mL^−1^.

The morphology of the electrodeposited ZnO films is shown in Figure 5. A rod-like morphology is obtained for all films. Rod density and size varied as a function of GO content and electrodeposition method, namely they increased when the nucleation deposition step was applied. When GO was employed, lower content resulted in lower rod dimensions and higher density, which is attributed to improved electrodeposition due to the availability of nucleation sites.

**Figure 6:**
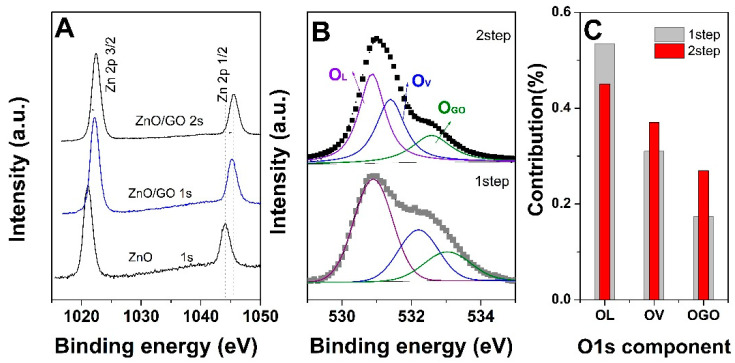
XPS Zn 2p core level spectra (**A**), deconvoluted O 1s core level spectra (**B**) corresponding contribution to the O 1s of deconvoluted components (**C**) for ZnO electrodeposited onto GO 0.1 mg mL^−1^ as a function of method.

XPS measurements were performed to explore the surface chemistry of the ZnO electrodeposited onto the GO nucleation layer as a function of the electrodeposition mode and GO content. Figure 6A depicts the evolution of high-resolution Zn 2p spectra. The typical spin–orbit doublets of 2p states were observed at 1044 and 1021 eV, respectively, and are attributed to binding energies of the Zn 2p1/2 and Zn 2p3/2 electronic states [30]. The peak separation between the binding energies corresponds to the standard reference value for ZnO, indicative of +2 valence state for Zn in wurtzite ZnO [31]. The Zn 2p spectra showed a shift in the binding energies to a high energy region (1045.3 eV and 1022.2 eV) for ZnO electrodeposited by the 1-step method onto GO-based nucleation layer (0.1 mg mL^−1^) as compared to the absence of GO. The application of a nucleation step further shifted the binding energy to 1045.44 and 1022.37 eV. Such shifts in binding energy confirm a good interaction between the ZnO and GO layer [32].

Figure 5B depicts the O 1s core level spectra of ZnO electrodeposited onto GO 0.1 mg mL^−1^ as a function of deposition method. The peak was deconvoluted into three components using Gaussian fitting with the subtraction of a Shirley-type background. The O_L_ peak centered at the lowest binding energy of 530.86 eV is attributed to the lattice oxygen (Zn-O bond) of the ZnO matrix. The O_V_ component assigned to oxygen vacancies in the ZnO matrix [33] is located at a binding energy of 531–532 eV and represents defects in the ZnO lattice. The O_GO_ component observed at 532.6–533 eV is assigned to the interface interaction between ZnO and the GO layer [34]. To estimate the contribution of oxygen vacancies and hydroxides, the ratio of the oxygen component areas was depicted in Figure 6C. One can observe that the application of a nucleation step on GO in a low content results in an increase of O_V_ and O_GO_ components due to the improved interaction of ZnO with GO at higher cathodic potential.

**Figure 7:**
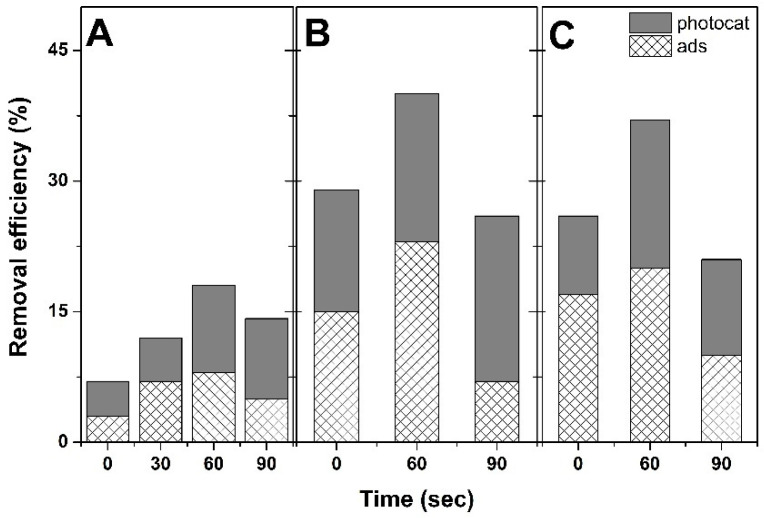
Contribution to removal efficiency for ZnO with GO concentration (mg mL^−1^): 0 (**A**), 0.1 (**B**), and 0.25 (**C**).

The photocatalytic properties were further investigated as a function of the deposition method and GO content, as depicted in Figure 7. The contribution of the adsorption (dark measurements) and photocatalytic degradation (120 min of irradiation) on the total removal efficiency is presented. Considering the adsorption, Figure 7 shows that the nucleation step aids in improving it up to a maximum duration corresponding to 60 s, regardless of using GO or not. A 5-fold MB adsorption was achieved in the presence of GO with respect to ZnO obtained without GO by the 1-step method. By including the nucleation step, a further increase in adsorption was obtained, clearer for the lower content of GO. However, a longer nucleation appeared detrimental for further improving the adsorption properties. The adsorption results could be explained by the nanostructure’s density, that can be tailored by nucleation conditions, and which affects the active surface area of the immobilized nanostructured photocatalyst. Thus, an increase in the density of nanostructures of lower dimensions will result in a higher active area and as such, both dye adsorption and the availability of sites for photocatalytic degradation will be improved. Conversely, a lower active surface area exhibits fewer active sites on the surface, thus, lower performance [35]. The photocatalytic ability of the ZnO obtained by the 1-step method appeared markedly improved in the presence of a lower content of GO. By applying a nucleation step, a further increase in photocatalytic contribution was achieved with nucleation duration in the case of a lower content of GO. A higher content of GO showed a maximum of both adsorption and photocatalytic activity up to a maximum duration of 60 s. These results could be explained by the role of functional groups in GO as nucleation sites for the electrodeposition of ZnO and are in line with reported research [36], being indicative of the marked effect of GO content on the adsorption [37] and photocatalytic properties [38].

**Figure 8:**
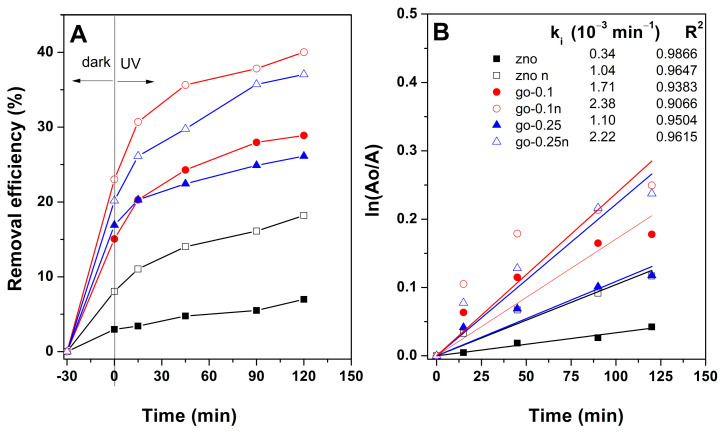
Kinetics of MB removal on ZnO films (**A**). First-order photodegradation kinetics including rate constant k (**B**).

To provide an insight into the MB photodegradation kinetics on the electrodeposited ZnO films, Figure 8 depicts the fitting according to first-order model. The fitting correlation R-square above 0.9, confirming photodegradation, followed the first-order kinetic model in all cases [39]. The kinetic apparent rate constant was increased when nucleation (60 s) was applied on the GO layer, being higher for a lower GO concentration. Thus, an increase in rate constant up to 2.38 × 10^−3^ min^− 1^, corresponding to 2-step method on GO 0.1, was achieved. This performance represents a 7-fold with respect to ZnO in the absence of GO by the 1-step method.

The obtained results are explained by the size and density of nanostructures in the obtained ZnO films as a function of GO and the additional deposition step [40]; namely, a smaller size and higher density of structures lead to a thinner film, thus a higher light absorption and delayed recombination of photogenerated carriers due to their enhanced transport [27]. The photocatalytic activity of ZnO nanorods was also reported to increase with the oxygen defects [41] by electron trapping, thus reducing the recombination rate of photo-generated charge carriers [42]. As both adsorption and photocatalytic ability improved for ZnO electrodeposited with an additional step on the GO layer, a stronger interface between ZnO and GO was obtained, similarly to other carbon nanomaterials [43].

While the photocatalytic degradation conditions in terms of the exposed surface area of the immobilized photocatalyst, light source and irradiance, deposition method, and dye concentration do now allow a straightforward comparison of the obtained results with the reported literature, Table 1 illustrates that the obtained results fall in the same range of performance and thus the relevance of the presented approach for such an application is confirmed.

Considering the importance of catalyst loading on the number of active sites for dye degradation, to further increase the performance of the proposed ZnO-GO, the size of the exposed area of the photocatalyst could be adjusted along with the irradiation conditions. The proposed supported ZnO-GO hybrid photocatalyst presents the advantages of time and cost-efficient recycling. Similar hybrids were reported to exhibit good performance retention upon four photocatalytic cycles [48].

In regard to the photocatalytic mechanism for dye degradation, scavenger measurements of photocatalytic degradation on similar hybrids indicated that hydroxyl radicals had a major role in the MB photodegradation [6]. Under irradiation, ZnO absorbs photons, and electron–hole pairs are generated. These pairs reach the ZnO surface to participate in the degradation reactions. The holes in the valence band react with water molecules to result in hydroxyl radicals that further attack the MB molecules adsorbed at the ZnO surface, while the electrons in the conduction band transfer to the electroreduced GO, which acts as an electron trap, hindering the charge recombination. Therefore, the photocatalytic degradation is improved. A further increase can be achieved by tailoring the distribution of the ZnO nanostructures and their size and structure by employing a low amount of GO, a buffer layer, and a nucleation step towards the electrodeposition of ZnO nanostructures. While the obtained hybrids exhibited significant adsorption in the dark, the overall removal performance appears improved. Such an aspect could be tailored by adjusting the GO amount in the buffer layer and the exposed surface area of the photocatalyst. The GO amount is observed to result in a performance increase in the lower range, this being attributed to an improved distribution of GO sheets in the buffer layer, thus exposing the decorating groups as nucleation sites for ZnO.

## 4. Conclusions

ZnO nanostructured films were electrodeposited in varying conditions towards improving the nucleation process by exploiting functional groups in GO as nucleation sites and an additional electrodeposition step at a greater cathodic potential. The obtained films were shown to exhibit improved adsorption and photocatalytic activity with a marked effect of nucleation duration and GO content. Optimum adsorption and photocatalytic ability were obtained for a nucleation duration of 60 s on GO (in about 50:50% contribution). The highest MB removal efficiency was 40% and the photodegradation rate constant was 2.38 × 10^−3^ min^−1^, representing about 6-folds and 7-folds value with respect to the counterparts obtained without nucleation condition adjustments. The obtained results indicate the GO and an additional electrodeposition step in potentiostatic mode could be successfully employed to improve ZnO adsorption and photocatalytic activity for dye removal in residual waters.

## Figures and Tables

**Table 1:** Comparison of photocatalytic degradation performance (efficiency, η and rate constant, k) of supported ZnO-GO hybrid photocatalysts.

Material, Exposed Surface	Electrodeposition Conditions	Electrolyte, Concentration	Analyte, Concentration	Irradiation (Source, Irradiance, Exposure Duration)	k, 10^−3^ min^−1^	η, %	Ref.
ZnO 6.25 cm^2^	Sol–gel		MB 14.8 µM	120 W, 24 h	0.37	42	[44]
ZnO 1 cm^2^	magnetron sputtering	-	MB 1 µM	8 W UV lamp, 1.86 mW/cm^2^, 540 min	1	64	[14]
ZnO 4 cm^2^	−0.8 V	10 mM Zn(NO_3_)_2_	MB 62 µM	1000 W Xe/Hg lamp, 4 h		55	[45]
ZnO–rGO 4 cm^2^	−1.1 V, 1 min/ −1 V, 45 min	50 mM Zn(NO_3_)_2_ + 0.1 M KCl + 5 mg/L GO	Ofloxacin 5 mg/L	4 W Xe lamp 0.4 mW/cm^2^, 180 min	2.24	35	[46]
ZnO/G on Ni foam	−0.8 V, 60 min	10 mM Zn(NO_3_)_2_ + 0.1 M KCl + 0.1 M KNO_3_	MB 4 µM	UV, 8 W, 165 min		57	[47]
ZnO-GO 1 cm^2^	−1.3 V, 0.5 min/ −1.05 V, 10 min	10 mM Zn(NO_3_)_2_, 0.1 mg/L GO	MB, 1 µM	4 W, UV lamp, 0.4 mW/cm^2^, 90 min	2.38	40	This work

## Data Availability

The data that support the findings of this study are available from the corresponding author upon reasonable request due to privacy.
